# Supplementary material for: Proportion and distribution of neurotransmitter-defined cell types in the ventral tegmental area and substantia nigra pars compacta
Source: Addict Neurosci. Author manuscript; Available in PMC 2025 May 22. (PMC12097539; doi:10.1016/j.addicn.2024.100183)
Supplement: 1 [file NIHMS2080654-supplement-1.pdf]

Bregma (mm)	TPH2+ neuron counts	VMAT2+ neuron counts	Percentage of VMAT2+ neurons positive for TPH2	Mean percent for bregma point
**-3.6**	2	135	1.5	3.1
	2	127	1.6	
	7	94	7.4	
**-3.7**	13	109	11.9	9.0
	4	80	5	
**-3.9**	4	73	8.2	8.2
**-4.0**	2	24	8.3	7.0
	2	33	6.1	
**-4.1**	4	16	25	29.6
	4	11	36.4	
**-4.2**	13	27	48.1	48.1

Supplemental Table 1 (related to Figure 2). Fraction of TPH2/VMAT2 copositive neurons in caudal VTA (unilateral).

Bregma (mm)	Region	ZsG+ neuron counts	ZsG+ neurons with TH	Percent of co-positive neurons
-3.0	VTA	146	46	31.5
-3.1	VTA	182	31	17
-3.2	VTA	221	45	20.4
-3.4	VTA	187	31	16.6
-3.4	VTA	111	15	13.5
-3.5	VTA	131	17	13
-3.7	VTA	154	4	2.6
-3.7	VTA	153	0	0
-3.8	VTA	146	2	1.4
-3.9	VTA	122	4	3.3
-3.9	VTA	110	0	0
-4.1	VTA	17	1	5.9
-3.0	SNc	76	30	39.5
-3.1	SNc	79	18	22.8
-3.2	SNc	101	20	19.8
-3.4	SNc	82	17	20.7
-3.4	SNc	138	21	15.2
-3.5	SNc	146	18	12.3
-3.7	SNc	123	18	14.6
-3.7	SNc	113	6	5.3
-3.8	SNc	92	7	7.6
-3.9	SNc	110	5	4.5
-3.9	SNc	34	1	2.9

Supplemental Table 2 (related to Figure 4, Supplemental Figure 3). Fraction of ZsG/TH copositive neurons in VTA and SNc of VGAT-Cre reporter mice.

		n (mice)	n (sections)	Sex	VMAT2 only	VGAT only	VGLUT2 only	VMAT2$^+$/ VGAT$^+$	VMAT2$^+$/ VGLUT2$^+$	VGAT$^+$/ VGLUT2$^+$	Triple	Total
VTA global	Cumulative cell count	3	25	M	2597	2417	1906	166	871	771	139	8867
		3	23	F	2716	1871	1865	129	585	654	102	7922
		6	48	B	5313	4288	3771	295	1456	1425	241	16789
	Fraction (%)	3	25	M	29.3	27.3	21.5	1.9	9.8	8.7	1.6	-
		3	23	F	34.3	23.6	23.5	1.6	7.4	8.3	1.3	-
		6	48	B	31.6	25.5	22.5	1.8	8.7	8.5	1.4	-
PIF	Cumulative cell count	2	7	M	221	63	60	2	41	24	5	416
		2	7	F	199	46	121	4	24	20	2	416
		4	14	B	420	109	181	6	65	44	7	832
	Fraction (%)	2	7	M	53.1	15.1	14.4	0.5	9.9	5.8	1.2	-
		2	7	F	47.8	11.1	29.1	1.0	5.8	4.8	0.5	-
		4	14	B	50.5	13.1	21.8	0.7	7.8	5.3	0.8	-
PN	Cumulative cell count	2	9	M	254	211	176	10	173	26	19	869
		2	9	F	324	150	171	8	124	36	5	818
		4	18	B	578	361	347	18	297	62	24	1687
	Fraction (%)	2	9	M	29.2	24.3	20.3	1.2	19.9	3.0	2.2	-
		2	9	F	39.6	18.3	20.9	1.0	15.2	4.4	0.6	-
		4	18	B	34.3	21.4	20.6	1.1	17.6	3.7	1.4	-
PBP	Cumulative cell count	3	22	M	1824	1552	678	135	387	321	63	4960
		3	21	F	1965	1332	679	102	267	282	54	4681
		6	43	B	3789	2884	1357	237	654	603	117	9641
	Fraction (%)	3	22	M	36.8	31.3	13.7	2.7	7.8	6.5	1.3	-
		3	21	F	42.0	28.5	14.5	2.2	5.7	6.0	1.2	-
		6	43	B	39.3	29.9	14.1	2.5	6.8	6.3	1.2	-
RLi	Cumulative cell count	3	13	M	49	47	548	4	55	220	17	940
		2	11	F	8	36	473	2	26	138	10	693
		5	24	B	57	83	1021	6	81	358	27	1633
	Fraction (%)	3	13	M	5.2	5.0	58.3	0.4	5.9	23.4	1.8	-
		2	11	F	1.2	5.2	68.3	0.3	3.8	19.9	1.4	-
		5	24	B	3.5	5.1	62.5	0.4	5.0	21.9	1.7	-
IF	Cumulative cell count	2	10	M	58	39	197	5	81	98	22	500
		2	10	F	88	70	199	8	66	127	23	581
		4	20	B	146	109	396	13	147	225	45	1081
	Fraction (%)	2	10	M	11.6	7.8	39.4	1.0	16.2	19.6	4.4	-
		2	10	F	15.1	12.0	34.3	1.4	11.4	21.9	4.0	-
		4	20	B	13.5	10.1	36.6	1.2	13.6	20.8	4.2	-
VTAR	Cumulative cell count	3	6	M	95	77	104	4	12	45	0	337
		2	7	F	86	45	146	1	14	36	1	329
		5	13	B	181	122	250	5	26	81	1	666
	Fraction (%)	3	6	M	28.2	22.8	30.9	1.2	3.6	13.4	0	-
		2	7	F	26.1	13.7	44.4	0.3	4.3	10.9	0.3	-
		5	13	B	27.2	18.3	37.5	0.8	3.9	12.2	0.2	-
CLi	Cumulative cell count	3	8	M	96	428	143	6	122	37	13	845
		2	5	F	46	192	76	4	64	15	7	404
		5	13	B	142	620	219	10	186	52	20	1249
	Fraction (%)	3	8	M	11.4	50.7	16.9	0.7	14.4	4.4	1.5	-
		2	5	F	11.4	47.5	18.8	1.0	15.8	3.7	1.7	-
		5	13	B	11.4	49.6	17.5	0.8	14.9	4.2	1.6	-
SNc global	Cumulative cell count	3	22	M	1845	1681	412	179	219	22	25	4383
		3	21	F	1749	1319	345	158	212	16	28	3827
		6	43	B	3594	3000	757	337	431	38	53	8210
	Fraction (%)	3	22	M	42.1	38.4	9.4	4.1	5.0	0.5	0.6	-
		3	21	F	45.7	34.5	9.0	4.1	5.5	0.4	0.7	-
		6	43	B	43.8	36.5	9.2	4.1	5.2	0.5	0.6	-
SNcD	Cumulative cell count	3	21	M	1133	938	221	150	130	11	4	2587
		2	20	F	1126	705	230	128	156	10	11	2366
		5	41	B	2259	1643	451	278	286	21	15	4953
	Fraction (%)	3	21	M	43.8	36.3	8.5	5.8	5.0	0.4	0.2	-
		2	20	F	47.6	29.8	9.7	5.4	6.6	0.4	0.5	-
		5	41	B	45.6	33.2	9.1	5.6	5.8	0.4	0.3	-
SNcL	Cumulative cell count	3	18	M	82	330	171	9	64	11	21	688
		3	16	F	59	246	89	8	47	6	17	472
		6	34	B	141	576	260	17	111	17	38	1160
	Fraction (%)	3	18	M	11.9	48.0	24.9	1.3	9.3	1.6	3.1	-
		3	16	F	12.5	52.1	18.9	1.7	10.0	1.3	3.6	-
		6	34	B	12.2	49.7	22.4	1.5	9.6	1.5	3.3	-
SNcM	Cumulative cell count	3	15	M	571	317	18	19	12	0	0	937
		2	13	F	510	245	26	19	6	0	0	806
		5	28	B	1081	562	44	38	18	0	0	1743
	Fraction (%)	3	15	M	60.9	33.8	1.9	2.0	1.3	0	0	-
		2	13	F	63.3	30.4	3.2	2.4	0.7	0	0	-
		5	28	B	62.0	32.2	2.5	2.2	1.0	0	0	-
SNcV	Cumulative cell count	3	9	M	59	96	2	1	13	0	0	171
		2	9	F	54	123	0	3	3	0	0	183
		5	18	B	113	219	2	4	16	0	0	354
	Fraction (%)	3	9	M	34.5	56.1	1.2	0.6	7.6	0	0	-
		2	9	F	29.5	67.2	0.0	1.6	1.6	0	0	-
		5	18	B	31.9	61.9	0.6	1.1	4.5	0	0	-

Supplemental Table 3 (related to Figures 4 & 5). Cell-type counts and fractions for subregions disaggregated by sex.

Experiment	Label	Laser λ (nm)	Laser source	Laser Power	Gain (ms)
Slc17a6*/*Slc32a1*/*Slc18a2	DAPI	405	Diode	0.50%	611
	Slc17a6	488	Argon	0.50%	558
	Slc32a1	561	Diode-pumped solid-state	0.50%	750
	Slc18a2	633	Helium-Neon	0.50%	700
**Dual ISH and IHC**	DAPI	405	Diode	0.50%	611
	GFP	488	Argon	0.15%	558
	Slc17a6	561	Diode-pumped solid-state	0.50%	750
**TPH2**	DAPI	405	Diode	0.50%	611
	Slc18a2	488	Argon	0.40%	558
	Tph2	561	Diode-pumped solid-state	0.18%	750
	Th	633	Helium-Neon	0.50%	700

Supplemental Table 4 (related to methods). Image acquisition settings.

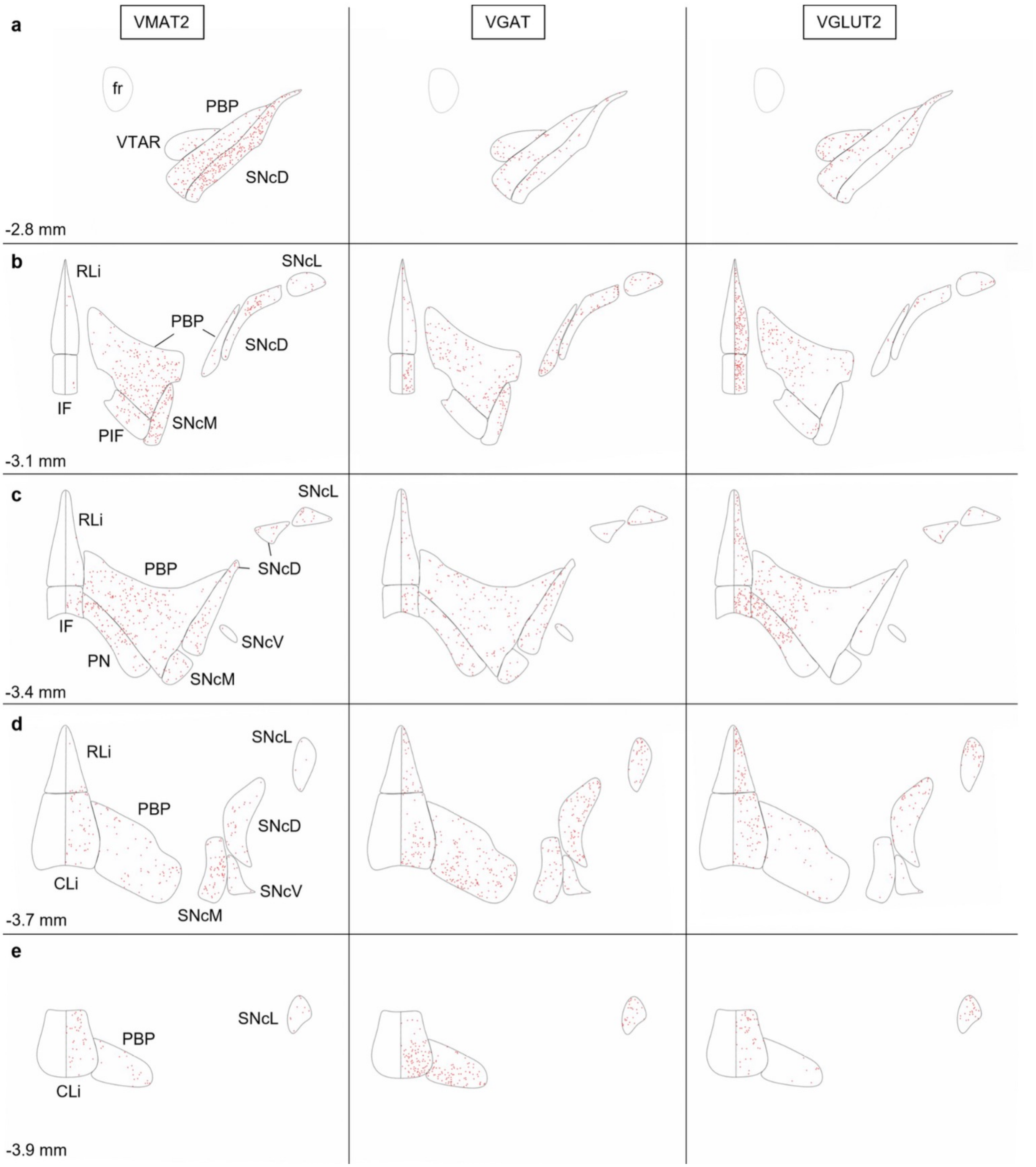

Supplemental Figure 1 (related to Figure 1). Schematized display of vesicular transporter-expressing neurons. (**A-E**) Each plot is from the corresponding image in **Figure 1** and each red dot represents the location of a counted VMAT2⁺, VGAT⁺, or VGLUT2⁺ neuron. CLi (caudal linear nucleus), fr (fasciculus retroflexus), IF (interfascicular nucleus), PBP (parabrachial pigmented nucleus), PIF (parainterfascicular nucleus), PN (paranigral nucleus), RLi (rostral nucleus), SNcD (SNc dorsal), SNcL (SNc lateral), SNcM (SNc medial), SNcV (SNc ventral), VTAR (VTA, rostral).

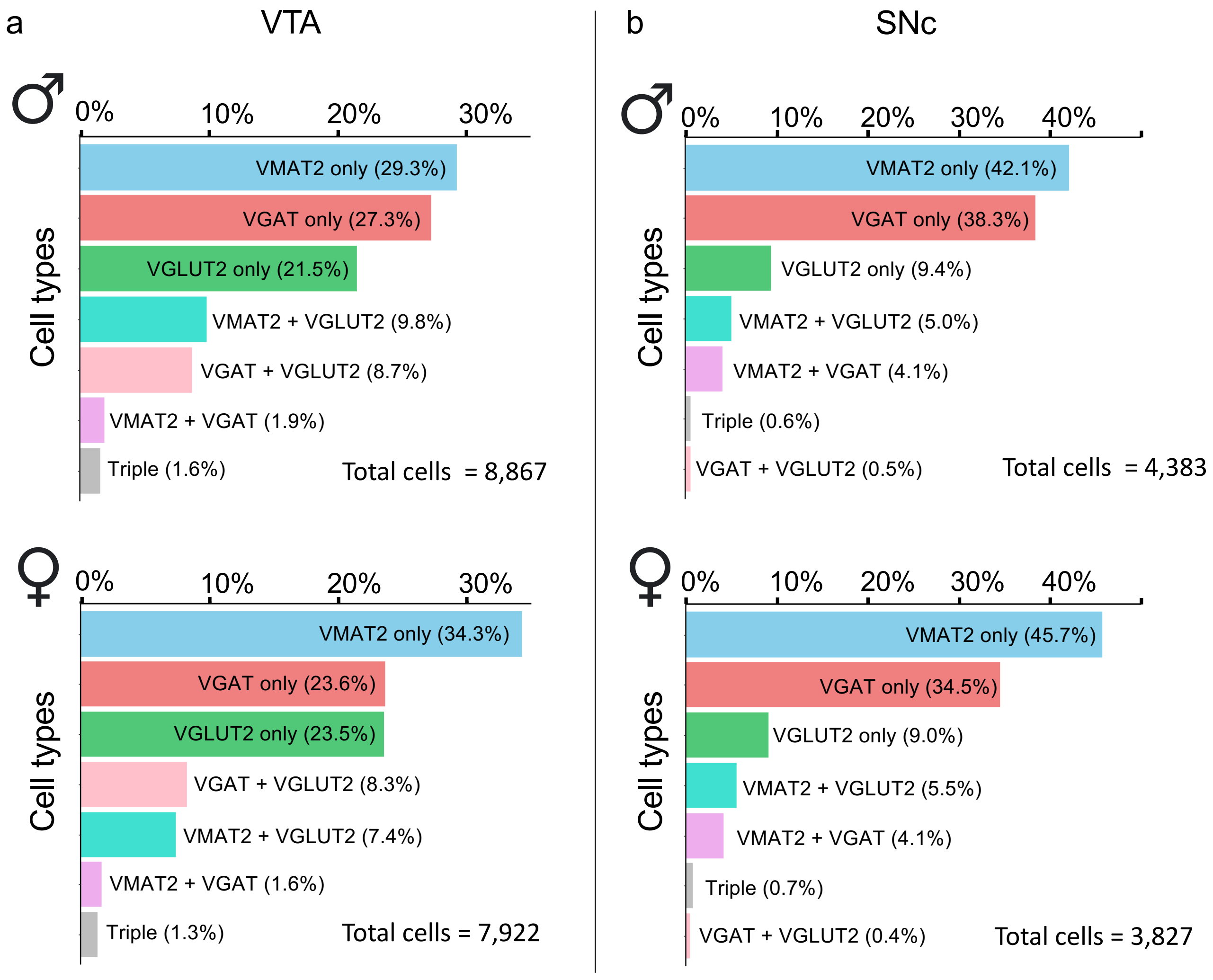

Supplemental Figure 2 (related to Figure 4). Global proportions of vesicular transporter-defined neurons in VTA and SNc displayed by sex. (A) Fraction of labeled VTA neurons that expressed one or more vesicular transporter in VTA of male (top panel) and female (bottom panel) mice. (**B**) Same as (A) but for SNc.

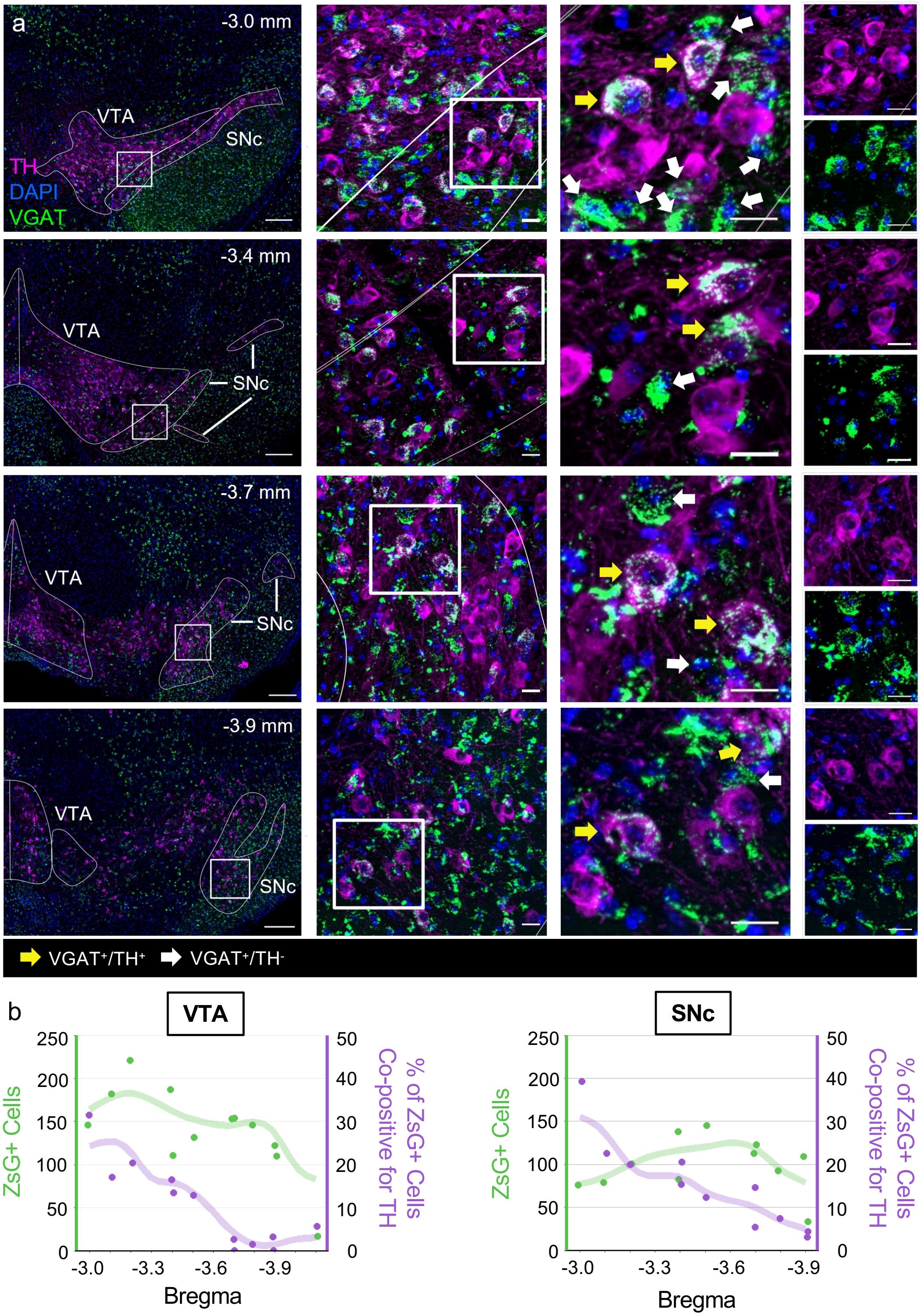

Supplemental Figure 3 (related to Figure 4). ZsG/TH co-positive neurons in VTA and SNc of VGAT-Cre reporter mice. (A) Example images of coronal sections from VGAT-Cre x ZsGreen reporter (green) mice immunostained for TH (magenta), with DAPI (indigo). Left panels show wide-field view with VTA and SNc demarcated, scale 200 μM. Middle left panels (scale, 20 μM) represent white box from left panel; middle right panels (scale, 20 μM) represent white box from middle left panel. Yellow arrows point to neurons co-positive for ZsG and TH, white arrows point to ZsG+ neurons negative for TH. Right panels separately show TH (top) and ZsG (bottom) signal from middle right panels. **(B)** Cell counts for ZsG+ cells and percentage of ZsG+ cells co-positive for TH by Bregma. Trend lines are moving averages smoothed with spline regression.

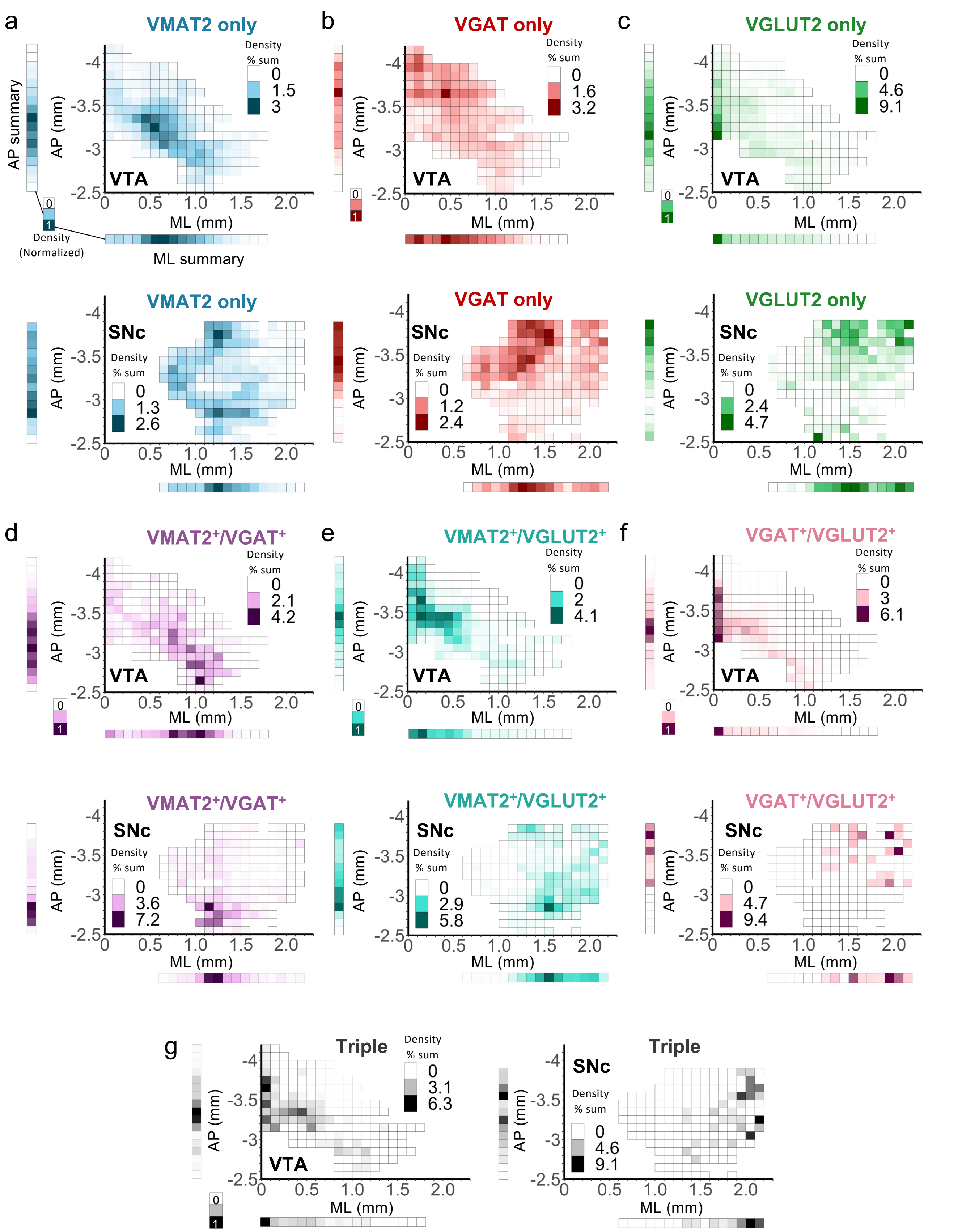

Supplemental Figure 4 (related to Figure 6). Spatial distribution of vesicular transporter-defined neurons along anterior-posterior (AP) and medial-lateral (ML) axes. Density heatmaps of neurons expressing (**A**) VMAT2-only, (**B**) VGAT-only, (**C**) VGLUT2-only, (**D**) VMAT2$^+$/VGAT$^+$, (**E**) VMAT2$^+$/VGLUT2$^+$, (**F**) VGAT$^+$/VGLUT2$^+$ or (**G**) all three vesicular transporters (Triple) across AP and ML axes. Summary bars display density data collapsed into either axis then normalized.
